# lncRNA Profiling of Exosomes and Its Communication Role in Regulating Silica-Stimulated Macrophage Apoptosis and Fibroblast Activation

**DOI:** 10.3390/biom14020146

**Published:** 2024-01-24

**Authors:** Jiaqi Ban, Shuai Chang, Pengwei Ma, Xin Wang, Fangwei Liu

**Affiliations:** 1 Division of Pneumoconiosis, School of Public Health, China Medical University, Shenyang 110122, China; banjiaqi@gmc.edu.cn (J.B.); 2020120081@stu.cmu.edu.cn (S.C.); 2021120329@cmu.edu.cn (P.M.); 2 The Key Laboratory of Environmental Pollution Monitoring and Disease Control, School of Public Health, Ministry of Education, Guizhou Medical University, Guiyang 550025, China; 3 Tianjin Centers for Disease Control and Prevention, Tianjin 300011, China; wangxin6@tj.gov.cn; 4 Key Laboratory of Environmental Stress and Chronic Disease Control and Prevention, Ministry of Education, China Medical University, Shenyang 110122, China

**Keywords:** exosomes, long non-coding RNA, cell communication, silicosis, macrophages, fibroblasts

## Abstract

Long-term silica particle exposure leads to interstitial pulmonary inflammation and fibrosis, called silicosis. Silica-activated macrophages secrete a wide range of cytokines resulting in persistent inflammation. In addition, silica-stimulated activation of fibroblast is another checkpoint in the progression of silicosis. The pathogenesis after silica exposure is complex, involving intercellular communication and intracellular signaling pathway transduction, which was ignored previously. Exosomes are noteworthy because of their crucial role in intercellular communication by delivering bioactive substances, such as lncRNA. However, the expression profile of exosomal lncRNA in silicosis has not been reported yet. In this study, exosomes were isolated from the peripheral serum of silicosis patients or healthy donors. The exosomal lncRNAs were profiled using high-throughput sequencing technology. Target genes were predicted, and functional annotation was performed using differentially expressed lncRNAs. Eight aberrant expressed exosomal lncRNAs were considered to play a key role in the process of silicosis according to the OPLS-DA. Furthermore, the increased expression of lncRNA MSTRG.43085.16 was testified in vitro. Its target gene *PARP1* was critical in regulating apoptosis based on bioinformatics analysis. In addition, the effects of exosomes on macrophage apoptosis and fibroblast activation were checked based on a co-cultured system. Our findings suggested that upregulation of lncRNA MSTRG.43085.16 could regulate silica-induced macrophage apoptosis through elevating PARP1 expression, and promote fibroblast activation, implying that the exosomal lncRNA MSTRG.43085.16 might have potential as a biomarker for the early diagnosis of silicosis.

## 1. Introduction

Pulmonary fibrosis caused by environmental silica dust exposure, called silicosis, has been a serious threat to human health [1]. Thousands of silicosis cases have been reported annually in China in the past 20 years [2]. More than 53,000 people have died from silicosis in the U.S. in the past few decades [3]. Unfortunately, there is still a lack of biomarkers with high specificity that can be utilized as an early diagnosis because of the unclear pathogenesis in irreversible fibrosis. Two major steps in the progress of silicosis were persistent inflammation and interstitial fibrosis. Macrophages could be activated by SiO_2_ particles and secrete inflammatory cytokines and chemokines, which help in the continuous expansion of inflammation [4]. Stimulation of persistent inflammation promoted the differentiation of fibroblasts into myofibroblasts and the secretion of extracellular matrix, such as collagen, which was critical in the development of fibrosis [5]. Apoptosis has been testified to participate in both steps during the process of silicosis. Inhibition of macrophage apoptosis contributed to alleviating silicosis [6,7,8]. Regulation of apoptosis could affect the transdifferentiation of fibroblasts and alter the pathological course of silicosis [9,10]. However, existing studies paid much attention to the intracellular signaling during fibrosis, but ignored the intercellular communication between different cells, such as macrophages and fibroblasts.

Exosomes have become a great candidate for intercellular communication due to many properties, such as low clearance rate and the ability to cross biological barriers [11,12,13,14]. After being released, exosomes could be englobed and unload their “cargo” to transmit signals in receptor cells [12,15,16,17,18]. Exosomes carry versatile cargoes, including long noncoding RNA (lncRNA), a type of transcript with a length of more than 200nt [19]. Aberrant expression of lncRNA was associated with the pathologic process of many diseases, due to its effect on some miRNAs or target genes [20,21,22,23]. LncRNA ZEB1-AS1 mediated epithelial–mesenchymal transition through competitively binding miR-141-3p in idiopathic pulmonary fibrosis [24]. LncRNA-LFAR1 exacerbated liver fibrosis by activating TGF-β and Notch pathways [25]. However, the expression profile of exosomal lncRNA in silicosis patients is still unknown, and the role of exosomal lncRNA in the pathogenesis of silicosis has not been clarified.

In this study, exosomes were isolated from the peripheral serum of silicosis patients or healthy donors. The whole transcriptome sequencing was performed to profile RNA expression in the exosomes. Bioinformatics analysis was employed to screen the crucial lncRNA with differential expression and predict its target genes. Our data showed that 27 lncRNAs expressed aberrantly in the exosome of silicosis patients, of which lncRNA MSTRG.43085.16 increased dramatically. The up-regulation of lncRNA MSTRG.43085.16 and the down-regulation of its target gene *PARP1* were verified in both silica-treated THP-1 and its released exosome. Meanwhile, the effect of exosomes on THP-1 apoptosis and fibroblast activation was also checked in vitro.

## 2. Materials and Methods

### 2.1. Study Subjects 

From March 2019 to October 2019, 20 silicosis patients (stage 1) from Ninth Hospital of Shenyang City and 29 health workers from the Northern Heavy Industries Group were recruited. All subjects involved in this study were well informed and confirmed about the purpose of this study, and have signed informed consent. An amount of 5 mL of peripheral blood was obtained from every subject. The serum was separated to isolate the exosome. 

### 2.2. Exosome Isolation

A total of 2 mL mixed serum was rapidly thawed at 37 °C, and it was centrifuged at 2000× *g*, 4 °C for 30 min. The sample was transferred to a new centrifuge tube and centrifuged at 2000× *g* for 30 min at 4 °C. Afterward, the supernatant was collected and centrifuged at 12,000× *g*, 4 °C for 45 min to exclude the pellet. The supernatant was taken, filtered through a 0.45 μm filter, and the filtrate was collected. The raw exosomes, including multivesicular bodies, were collected via centrifuging the supernatant at 110,000× *g* for 70 min (70 Ti centrifuge rotor, Beckman Coulter Inc., Brea, CA, USA). The suspension was collected and added to a new ultracentrifuge tube, centrifuging for 70 min at 110,000× *g* and discarding the supernatant. After repeating this step one time, the pellet of exosomes at the bottom of the centrifuge tube was resuspended in (50 µL) PBS.

### 2.3. RNA Quantification and Qualification

RNA in serum or exosomes was extracted using Trizol or BIOG Exosome RNA Easy Kit (51082, Baidai, Changzhou, China). RNA concentration and purity were measured using the NanoDrop 2000 Spectrophotometer (Thermo Fisher Scientific, Wilmington, DE, USA). RNA integrity was assessed using the RNA Nano 6000 Assay Kit of the Agilent Bioanalyzer 2100 System (Agilent Technologies, Santa Clara, CA, USA).

### 2.4. Library Preparation for lncRNA Sequencing

Sequencing libraries were generated using a NEBNext Ultra directional RNA library prep kit for Illumina (NEB). The poly (A) containing mRNA molecules was purified using poly (T) oligo-attached magnetic beads. Purified libraries were quantified using a Qubit 3.0 fluorometer (Life Technologies, Carlsbad, CA, USA) and validated using an Agilent 2100 Bioanalyzer to confirm the insert size and calculate the mole concentration. Library preparations were sequenced on an Illumina Novaseq 6000 platform and paired-end reads were generated. Library construction and sequencing were performed (Biomarker Corporation, Beijing, China).

### 2.5. Differentially Expressed lncRNAs Identification

Differential expression analysis of two conditions/groups was performed using the DESeq R package (version: 4.1.1). DESeq provided statistical routines for determining differential expression in digital gene expression data using a model based on the negative binomial distribution. The resulting P-values were adjusted using Benjamini and Hochberg’s approach for controlling the false discovery rate. Genes with an adjusted *p*-value < 0.01 and an absolute value of log2 (fold change) > 1 found by DESeq were identified as differentially expressed. Before differential gene expression analysis, the read counts were adjusted by the edgeR program package (version: 3.15) through one scaling normalized factor for each sequenced library. Differential expression analysis of two samples was performed using the EBseq (2010) R package (version: 1.36.0). The resulting FDR (false discovery rate) was adjusted using the PPDE (posterior probability of being DE). The FDR < 0.05 and |log2 (Fold Change)| ≥ 1 were set as the threshold for significant differential expression. OPLS-DA was performed using SIMCA-P 14.1 software (Umetrics, Malmo, Sweden).

### 2.6. LncRNA Target Gene Prediction and Functional Annotation

LncRNAs regulate the expression of their neighboring genes, which are predicted mainly based on the position of the lncRNA concerning the gene, and the neighboring genes within 100 kb of the lncRNA are its cis-target genes. The trans-target genes of lncRNAs were predicted by the correlation between lncRNA and mRNA expression between samples.

Target gene functional annotation: Gene function was annotated based on the following databases: NR (non-redundant protein sequence database); Swiss-Prot (a manually annotated, non-redundant protein sequence database); GO (Gene Ontology database); COG (the database of Clusters of Orthologous Groups of proteins); KOG (the database of Clusters of Protein homology); Pfam (the database of Homologous protein family); and KEGG (the database of Kyoto Encyclopedia of Genes and Genomes). Target gene functional enrichment analysis: GO enrichment analysis Gene Ontology (GO): enrichment analysis of the target gene of differentially expressed lncRNAs was implemented by the ClusterProfiler R packages (version: 3.8); KEGG pathway enrichment analysis: ClusterProfiler R packages were used to find the KEGG pathway that was significantly enriched compared to the entire genome background.

### 2.7. Cell Culture and Treatment

MRC-5 (human lung fibroblasts cell line) or THP-1 (human leukemia monocyte cell line) were obtained from the Type Culture Collection of the Chinese Academy of Sciences (Shanghai, China), and maintained in RPMI Medium 1640 or MEM containing 10% Exo-depleted FBS (Inner Mongolia Opcel BiotechnologyCo., Ltd., Heilinger, Inner Mongolia, China), (100 µg/mL) streptomycin, and (100 U ml^−1^) penicillin in 5% CO_2_ incubator at 37 °C. THP-1 cells were cultured in the RPMI Medium 1640 and additionally added with 2-mercaptoethanol (0.05 mM) and phorbol-12-myristate-13-acetate (PMA, 50 nM). After incubating for 72 h, THP-1 cells were differentiated into macrophages. In this article, THP-1 represents differentiated THP1 cells (macrophages). RNA in cells or exosomes was extracted using Trizol or BIOG Exosome RNA Easy Kit (51082, Baidai, China).

To identify the role of exosomes in regulating macrophage apoptosis, the differentiated THP-1 was divided into four groups as follows: Control, SiO_2_, GW4869+SiO_2_, and DMSO+SiO_2_. THP-1 in GW4869+SiO_2_ was pre-treated with GW4869 (an inhibitor of exosome release, 1 μL/mL) for four hours [26]. Then 1 × 10^8^ THP-1 in SiO_2_, GW4869+SiO_2_, and DMSO+SiO_2_ were treated with SiO_2_ (100 µg/mL, Min-U-Sil 5, Sigma-Aldrich, St. Louis, MO, USA, Particulates size distribution: 97% < 5 μm diameter and 80% < 3 μm diameter; median diameter 1.4 μm) for 24 h, and the supernatant was extracted and added into untreated differentiated THP-1 for 24 h [27].

To check the role of the exosome in regulating the activation of fibroblast, the supernatant of THP-1 treated with SiO_2_ or saline was extracted for exosome isolation. MRC-5 was divided into four groups as follows: Control, Exo-con, Exo-SiO_2_, and Exo(SiO_2_)+ SiO_2_. MRC-5 in the control group was cultured without exosomes. MRC-5 in the Exo-con group and Exo-SiO_2_ group was cultured with the exosome from THP-1 treated with saline. MRC-5 in Exo(SiO_2_)+ SiO_2_ group was cultured with exosome from THP-1 treated with SiO_2_. MRC-5 in the Exo-SiO_2_ group and Exo(SiO_2_)+ SiO_2_ group were treated with SiO_2_ for 24 h. Saline was used as a control.

### 2.8. TUNEL Analysis

TUNEL detection kit (Elabscience, Wuhan, China) was used for TUNEL analysis. After the cells were treated, the supernatant was aspirated, and the cells were washed with PBS (pH 7.4). A detailed operation of TUNEL staining according to the manufacturer’s instructions. After the staining of TUNEL, the supernatant was aspirated, and the cells were washed with PBS (pH 7.4). After that, the DAPI nucleus was stained with mounting fluid containing DAPI. Take out the coverslip and rapidly mount it for observation. 

### 2.9. Western Blot 

The Cell Mitochondria Isolation Kit (Beyotime, Shanghai, China) was used to obtain the mitochondrial proteins. The lysates were measured by the Pierce BCA Protein Assay Kit (Beyotime) and quantified to 3 μg/μL. Proteins were loaded on an 8–12% SDS-polyacrylamide gel which was electrophoretically transferred to PVDF membranes (Millipore, Darmstadt, Germany) after electrophoresis. The membranes were incubated at 4 °C overnight after rapid blocking buffer (Sevenbio, Beijing, China). The following primary antibodies were used in this experiment: anti-PARP1 (Proteintech, Chicago, IL, USA, 1:10,000), and anti-ACTB (CST, 1:1000). The membranes were probed with a horseradish peroxidase-conjugated secondary antibody (CST, 1:2000) at room temperature for an hour. A chemiluminescence detection system was used to detect protein bands.

### 2.10. Real-Time Quantitative PCR (RT-qPCR) for lncRNA Measurement

LncRNA was isolated from macrophages and reversed using RNA isolation and reverse kit (TaKaRa, Kusatsu, Japan) as per the manufacturer’s instructions. RT-qPCR for lncRNA. RT-qPCR was performed using a StepOnePlus RT-PCR System (Applied Biosystems, Foster, CA, USA). The primer for RT-qPCR was listed as follows: lncRNA MSTRG43085.16 F:5′- CAGGTCAAAACTCCCGTGCT-3′, lncRNA MSTRG43085.16 R: 5′-GACGGGGTCTCGCTATGTTG-3′. *PARP1* F:5′-AAGATAGAGCGTGAAGGCGAATGC-3′, *PARP1* R:5′- GTATGGCAGTAGTTGGCACTCTTGG-3′. *GAPDH* F:5′-GCACCGTCAAGGCTGAGAAC-3′, *GAPDH* R: 5′-TGGTGAAGACGCCAGTGGA-3′.

### 2.11. Immunofluorescence

MRC-5 cells were fixed with 4% paraformaldehyde, then blocked by 5% BSA containing 0.2% Triton X-100 for 30 min. The cells were incubated with primary antibodies α-SMA (CST, 1:200) overnight. FITC-conjugated secondary antibodies (1:100) were incubated at room temperature for an hour in darkness. The representative field of immunofluorescent images was captured using a confocal microscope. Nuclei were marked by DAPI.

### 2.12. Statistical Analysis

SPSS 21.0 was used for statistical analysis. A *t*-test was performed to analyze the difference between the two groups. *p* < 0.05 was considered to be statistically significant. All the data were shown as mean ± standard error of the mean (SEM).

## 3. Results

### 3.1. Baseline Characteristics of Silicosis Patients and Healthy Controls

Silicosis patients (diagnosed with stage 1) and health workers were recruited in this study. Their baseline characteristics was shown in Table 1. A total of 2 mL peripheral blood serum was used to extract exosomes for subsequent sequencing screening.

### 3.2. Identification of Differentially Expressed lncRNAs in Peripheral Blood Serum Exosomes of Silicosis Patients

**Figure 1:**
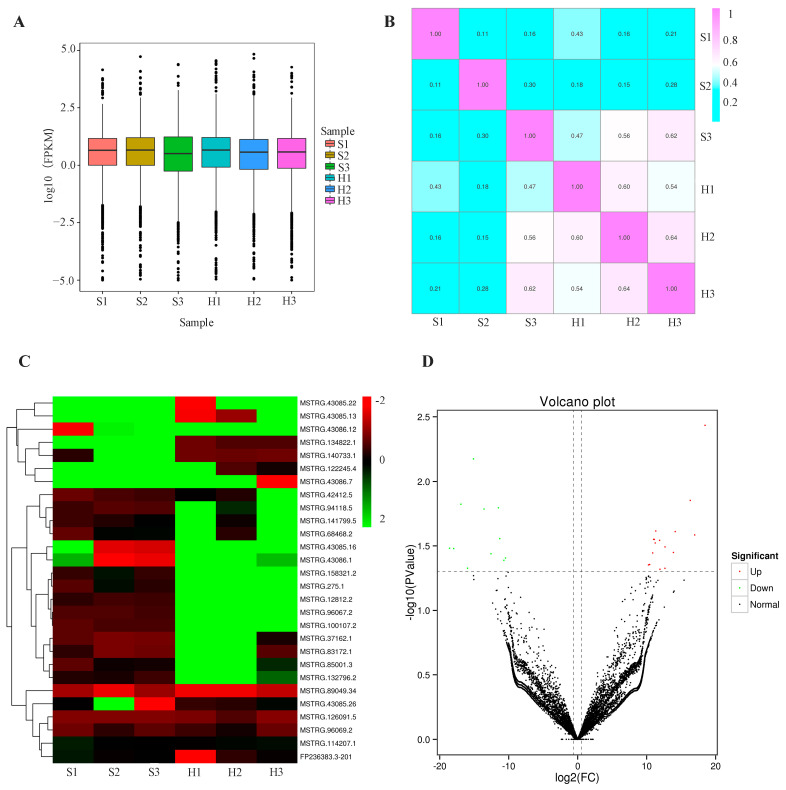
Differentially expressed lncRNAs were identified using the DESeq method. (**A**) The box plot was used to show the variance of each sample. The panels (**B**) represented the Pearson correlation analyses, respectively, which were used to investigate the coherence of the data distribution with the actual sample assignments. (**C**) The heat map was used to show the expression of different lncRNAs (red, upregulated; green, downregulated) and the clusters of different samples (H, the healthy control; S, the patient). (**D**) The volcano plot was used to identify differentially expressed lncRNAs with dots in different colors (red, upregulated; green, downregulated; and black, no significant difference).

A total of 12,055 lncRNAs were identified through the sequence alignment with the Ensemble database, of which 2685 sequences were consistent with the mature lncRNA records in Ensembl. In addition, 9370 sequences could not be matched with any records and were identified as predicted lncRNAs. The variance (Figure 1A), the correlation (Figure 1B), and the principal component analysis (Figure S1) were checked and performed, indicating that the quality control of samples was well. The heat map demonstrated the difference in gene expression between the two groups of samples, and cluster analysis was performed (Figure 1C). A total of 27 lncRNAs was found to be significantly differentially expressed between the silicosis patients and healthy controls, including 16 lncRNAs with up-regulated expression and 11 lncRNAs with down-regulated expression (Figure 1D).

### 3.3. Identification of Differentially Expressed lncRNAs Based on OPLS-DA

**Figure 2:**
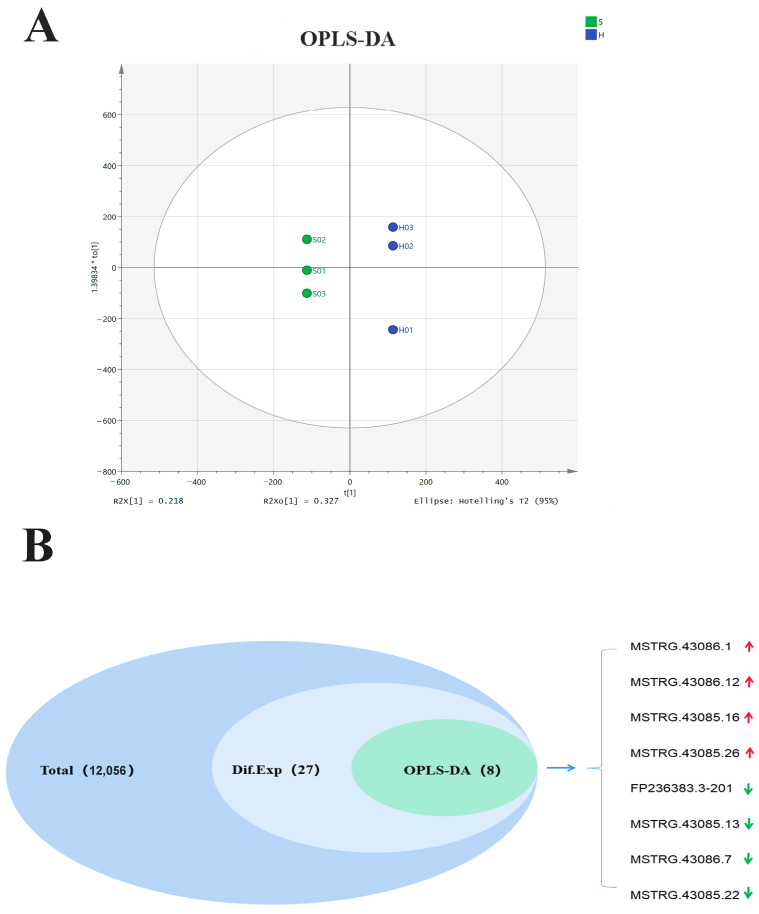
Differentially expressed lncRNAs were identified using the OPLS-DA method. (**A**) Clusters of all samples were analyzed using the OPLS-DA method (R2x ≈ 0.218, R2Xo ≈ 0.327). (**B**) The Venn diagram was used to show eight lncRNAs identified by DESeq and OPLS-DA (H, the healthy control group; S, the patient group; and Dif.Exp, differentially expressed lncRNAs; red arrow, four upregulated lncRNAs; green arrow, four downregulated lncRNAs).

Orthogonal Partial Least Squares Regression and Discriminant Analysis (OPLS-DA) was an algorithm to help identify unique metabolites among a large number of metabolites. Here, eight differentially expressed lncRNAs were identified using OPLS-DA, including seven predicted lncRNA and one mature lncRNA (Figure 2A,B, Table 2). All eight lncRNA were covered by the analysis results of the differential expression sequencing (DESeq) method, including MSTRG.43086.1, MSTRG.43086.12, MSTRG.43085.16, MSTRG.43085.26, FP236383.3-201, MSTRG.43085.13, MSTRG.43086.7, and MSTRG.43085.22 (Figure 2B). These results indicated a crucial role of these lncRNAs in the functional performance of exosomes.

### 3.4. Prediction and Functional Annotation of lncRNA Targets

**Figure 3:**
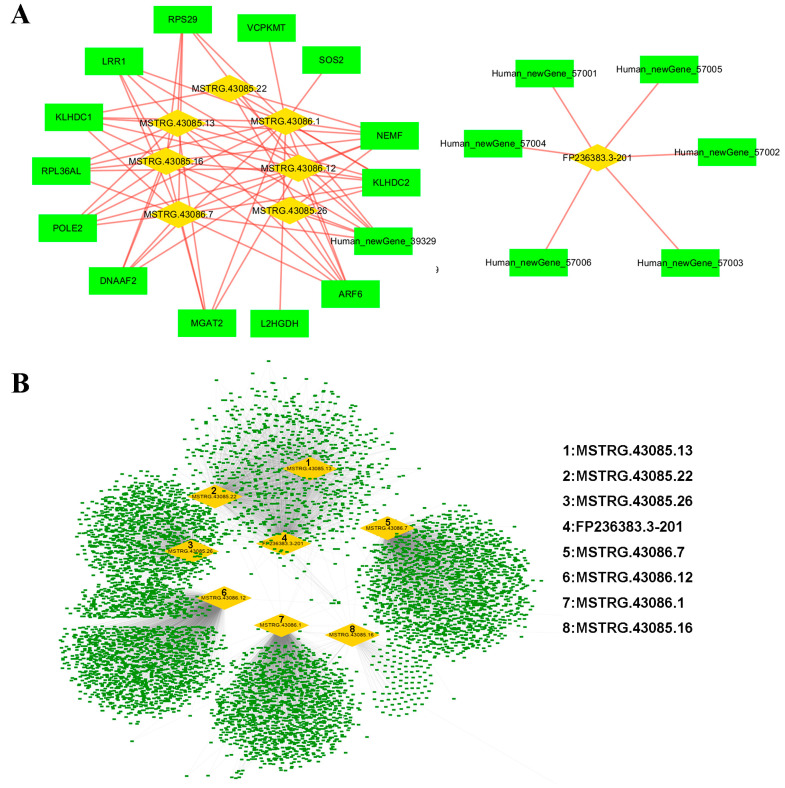
Target gene prediction of eight lncRNAs. A total of 68 Cis-target genes (**A**) and 8974 trans-target genes (**B**) corresponded to 8 distinctive lncRNAs. The Pearson correlation coefficient method was used to analyze the correlation between lncRNA and mRNA between samples, and genes with an absolute correlation value greater than 0.9 and a significant *p*-value less than 0.01 were used as the trans-target genes of lncRNA.

**Figure 4:**
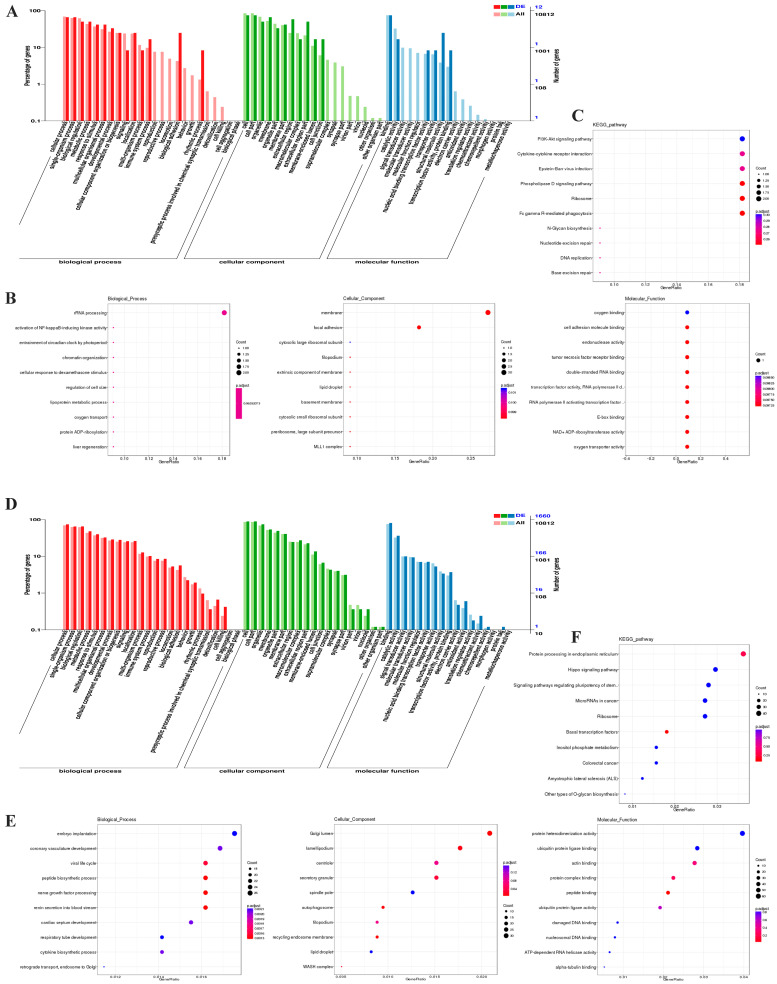
Functional annotation of 8 differentially expressed lncRNAs. (**A**,**B**) GO functional annotation of the cis-target genes of eight lncRNAs (indicated in Figure 2B). In addition, the top 10 GO items for biological process, molecular function, and cellular component. (**C**) KEGG pathway enrichment of the cis-target genes of eight lncRNAs. (**D**,**E**) GO functional annotation of the trans-target genes of eight lncRNAs. In addition, the top 10 GO items for biological process, molecular function, and cellular components. (**F**) KEGG pathway enrichment of trans-target genes of eight lncRNAs.

A total of 68 cis-target genes and 8974 trans-target genes of the eight lncRNAs were predicted according to their positions. Neighboring genes within 100 kb of lncRNA are usually considered to be their cis-target genes. The trans-target genes of lncRNA were identified by the Pearson correlation coefficient method (r > 0.9 and *p* < 0.01) (Figure 3). Next, the specific functions of these target genes of the 8 lncRNAs and their related signaling pathways need to be clarified. Thus, the functional annotation was performed based on the database of GO for both the cis-target genes and the trans-target genes. Three aspects of the target genes of lncRNAs were analyzed using the R package ClusterProfiler, including biological processes, molecular functions, and cellular components. The significantly enriched GO items were found using a hypergeometric test. In general, thirty-one GO second level items were enriched for the cis-target genes of (Figure 4A), and fifty-five for the trans-target genes (Figure 4D) distributed in all three aspects. Moreover, the top 10 GO items for each aspect were shown for either the cis-target genes (Figure 4B) or the trans-target genes (Figure 4E). Moreover, the enrichment of signaling pathways for the target genes was performed based on the KEGG database. The top 10 signaling pathways of the cis-target (Figure 4C) and trans-target (Figure 4F) genes of 8 lncRNAs enriched by KEGG analysis are also shown.

### 3.5. The Validation of lncRNA MSTRG.43085.16-PARP1 Regulatory Mechanism

**Figure 5:**
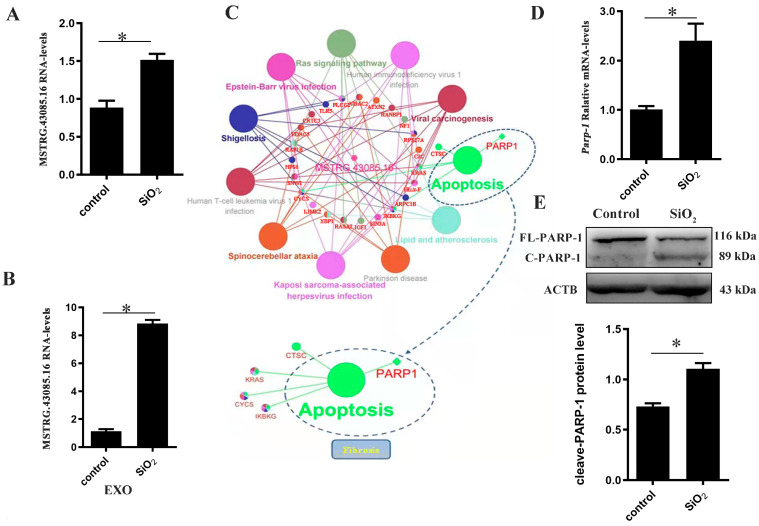
The expressions of lncRNA MSTRG.43085.16/PARP1 after silica treatment were verified. The expression of lncRNA MSTRG.43085.16 in silica-treated THP-1 (**A**) and exosomes isolated from the supernatant of silica-treated THP-1 (**B**) were checked using realtime-PCR. (**C**) Functional annotation of target genes corresponding to lncRNA MSTRG.43085.16. (**D**) The expression of *PARP1* in silica-treated THP-1 was checked using realtime-PCR. (**E**) The level of PARP1 in silica-treated THP-1 was measured using Western blot. Data were presented as mean ± SEM from at least three independent experiments. Asterisks indicated significantly different. * *p* < 0.05. Original images can be found in Supplementary Materials.

Next, we tried to verify the expressions of these 8 specific lncRNA through realtime-PCR assay in silica-treated macrophages. The expression of lncRNA MSTRG.43085.16 was significantly elevated in both the silica-treated macrophages and exosomes secreted by silica-treated macrophages (Figure 5A,B). Therefore, we reperformed functional annotation and pathway enrichment analysis of the target gene of lncRNA MSTRG.43085.16, the only one, which had a consistent expression change trend. Six aspects in A-class corresponding to twenty-five categories in B-class and fifty-five KEGG signaling pathways were demonstrated (Table S1). The apoptosis signaling pathway was identified in the functional re-annotation for all the trans-target genes of lncRNA MSTRG.43085.16 (Figure 5C). In addition, poly (ADP-ribose) polymerase 1 (PARP1), one of the target genes of lncRNA MSTRG.43085.16, showed a tight connection with multiple signaling pathways related to apoptosis (Table S2). The level of PARP1 was also elevated in silica-treated macrophages both at the gene level and at the protein level (Figure 5D,E).

**Figure 6:**
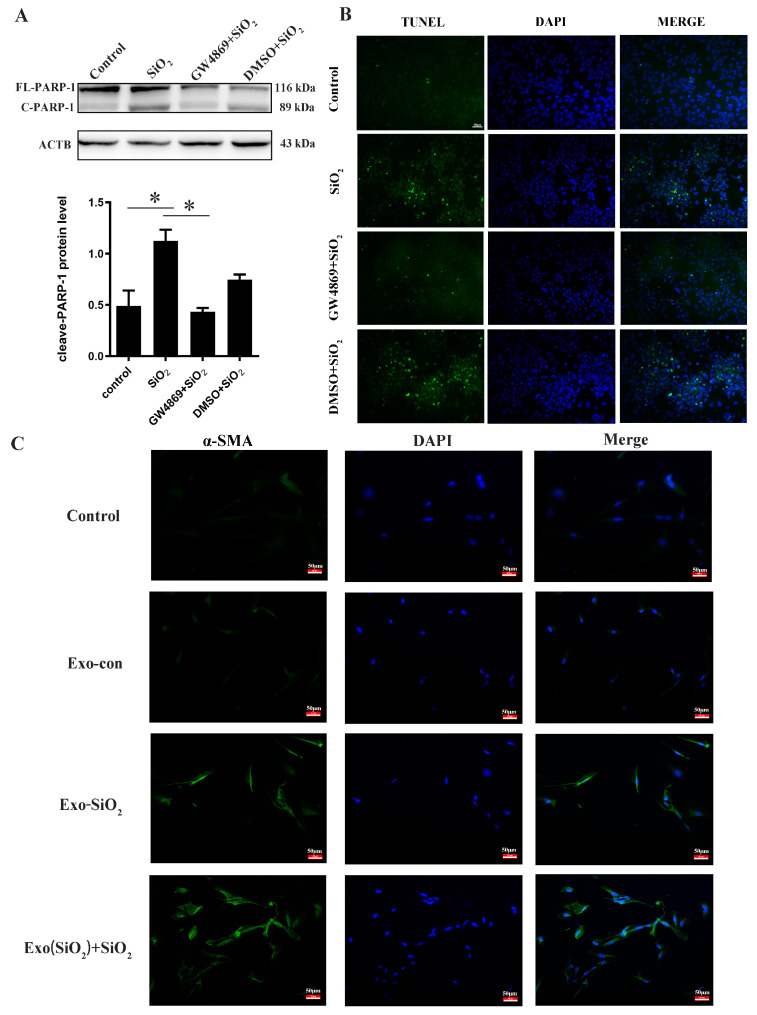
The role of exosome released from silica-treated THP-1 on macrophage apoptosis and fibroblast activation. To inhibit the release of exosomes, THP-1 was pre-treated with GW4869 for 4 hrs. followed by SiO_2_ treatment for 24 h. Then the supernatant with different treatments was transferred into untreated THP-1 as indicated for 24 h. The level of PARP1 (**A**) and the apoptotic cell death of THP-1 (**B**) were checked by Western blot and TUNEL assay. To check the effect of exosome on the expression of α-SMA, MRC-5 was cultured with exosome derived from THP-1 with (Exo(SiO_2_)) or without SiO_2_(Exo) stimulation, followed by SiO_2_ treatment for 24 h. The expression of α-SMA was detected using Immunofluorescence, the area in green color was indicated as a positive expression (**C**). Data were presented as mean ± SEM from at least three independent experiments. Asterisks indicated significantly different. * *p* < 0.05. Original images can be found in Supplementary Materials.

As the crucial defense in the pulmonary, the apoptosis of macrophages was known to be related to silica-induced lung inflammation and fibrosis [28]. Next, GW4869, an exosome inhibitor, was employed to show the effect of silica-induced exosomes on the apoptosis of THP-1 and the activation of MRC-5. As shown in Figure 6A, the level of PARP1 in the SiO_2_+GW4869 group was lower than that in the SiO_2_ group. Meanwhile, the regulation of silica-induced macrophage apoptosis was checked by TUNEL staining. The level of apoptosis was decreased in the SiO_2_+GW4869 group compared with that in the SiO_2_ group (Figure 6B). These data suggested that blocking the secretion of exosomes reduced the level of PARP1 and restricted apoptosis. On the other hand, the activation of fibroblasts could promote silica-induced collagen deposition and contribute to pulmonary fibrosis. Therefore, we studied the typical activation marker of fibroblasts, α-SMA. The exosome was purified from the supernatant of macrophages treated with or without silica and then added to MRC-5 fibroblasts. The immunofluorescence results showed that the level of α-SMA in the Exo-SiO_2_ group was significantly higher than that in the Exo-con group, which indicated that SiO_2_ could promote the expression of α-SMA. The fluorescence brightness was strongest in Exo(SiO_2_) + SiO_2_ groups, indicating that exosomes derived from SiO_2_-stimulated THP-1 could further amplify the promotion compared with that from unstimulated THP-1 (Figure 6C).

## 4. Discussion

This study recruited silicosis patients and healthy controls based on diagnostic criteria (GBZ 70-2015). There was no significant difference in age between silicosis patients and healthy controls, which could avoid the influence of age on the secretion of exosomes. Three patients and three health controls were chosen for the following sequencing based on the match conditions, including gender, age, exposure years, lifestyle, and other factors. It was also worth noting that the control group recruited in this study was composed of workers who were exposed to silica dust but had not developed the disease. The advantage of this choice was that the background of the two groups would be highly similar, such as work patterns, group diet, and other factors. However, there were some disadvantages, for example, the difference between the two groups might not be entirely caused by dust exposure. The genetic difference may also contribute. In addition, the difference in silica dust exposure duration between the two groups was statistically significant. The possible reason was that silicosis patients are removed from silica particle-exposure work once diagnosed. The exosome was isolated from 2 mL peripheral blood serum, preparing for the following sequencing. The poly (T) oligo-attached magnetic beads were applied to enrich the lncRNAs with polyA tail in this study. However, this conventional enrichment approach ran the risk of losing a small fraction of lncRNAs that did not have a polyA tail. The research progress of methodology showed that the rRNA depletion method could effectively avoid this situation [29].

The 27 lncRNAs with aberrant expression were identified by the whole transcriptome sequencing in the peripheral exosomes of silicosis patients. OPLS-DA was employed to analyze and narrow down the scale of differentially expressed lncRNA. Traditional analysis methods based on single gene expression level, including *t*-test, Wilcoxon rank-sum test, DESeq, and microarray linear model have obvious defects in analyzing genomic expression [30,31,32]. OPLS-DA was a comprehensive statistical method of univariate and multivariate statistical analysis and was usually used to determine unique substances in omics studies [33]. The obvious advantage of the OPLS-DA was that grouping information was taken into account while achieving dimension reduction. Thus, it was increasingly used to screen differentially expressed targets between different groups in multiple omics data [34]. In this study, we integrated DESeq and OPLS-DA to screen lncRNAs. Eight lncRNAs identified for the downstream analysis, with the best extent with the highest accuracy and specificity.

The number of exosomes released from the inflammatory cells, such as macrophages, increased two-to-three-fold in response to the exogenous stimulation, although about half of the exosomes locally in the lung are at steady-state and derived from epithelial cells [35]. Therefore, the expressions of those eight lncRNAs were checked by realtime-PCR in silica-treated macrophages, and only the lncRNA MSTRG.43085.16 was consistent with the sequencing results. Transcriptome sequencing, as a high-throughput sequencing method, is measured in the full-field region of a gene, while RT-PCR experiments require the design of a primer sequence to amplify a specific region of a gene, usually around 80–200 bp [36]. Therefore, the difference between RT-PCR and transcriptome sequencing in gene expression quantification can lead to inconsistency between the two in estimating the trend of the gene expression level. A certain degree of inconsistency between RT-PCR and RNA-seq (around 30–40%) is normal and reasonable, and the two cannot correspond to each other. Studies have shown that the correlation between RNA-seq and RT-PCR is around 0.8, 15.1–19.4% of RNA-seq results do not correspond to RT-PCR, and 1.6–2.8% of nonconcordant sequences are the exact opposite of RT-PCR results [27,37,38,39]. 

After lncRNA MSTRG.43085.16 became the focus of this study, we performed re-functional annotation (GO) and pathway enrichment (KEGG) analysis of its target genes, among which apoptosis drew a lot of attention. On the other hand, PARP1 was predicted to be the target gene of lncRNA MSTRG.43085.16 based on their relative position. Coincidentally, PARP1 was an important enzyme in regulating DNA replication [40]. PARP-1 depletion has been reported to protect from apoptosis and inhibit inflammatory signal transduction [41]. Inhibition of PARP1 has emerged as an effective anti-inflammatory intervention in many inflammatory-related diseases [42]. An existing study has shown an increased expression of PARP1 in SiO_2_-treated nonadherent cell lines [43]. Macrophages were known to play a critical role in the initiation and progression of silicosis, and the amount of exosome secretion was directly related to the size of the derived cell [4,12]. As a result, macrophages became our leading actor in the experiment in vitro. Our results showed that the expression of PARP1, the target gene of lncRNA MSTRG.43085.16, was increased in silica-treated macrophages. Furthermore, the effect of the exosome on the apoptosis of macrophages also was checked by TUNEL staining, which indicated that insufficient exosomes alleviated the level of apoptosis in macrophages. 

Fibroblast activation was known to contribute to irreversible pulmonary fibrosis, giving us a reason to consider fibroblasts were among the major recipient cells. As a result, the activated phenotype of fibroblasts, such as α-SMA was considered as a checkpoint to represent the fibrotic change. In order to show its communication role between macrophages and fibroblasts, exosomes were purified from the culture supernatant of macrophages with or without silica treatment. Fibroblasts were cultured with the extracted exosomes. The silica residue in exosomes could be effectively avoided because the exosome was isolated by the ultrahigh-speed centrifugation method. Moreover, the exosome size was 30–100 nm, as indicated previously [44,45], whereas the median diameter of the SiO_2_ particles used in the study was 1.4 μm, much larger than the size of exosomes. The results of immunofluorescence showed that macrophage-derived exosomes could activate fibroblasts, which indicated the crucial communicated role of exosomes carried with lncRNAs between macrophages and fibroblasts. Unfortunately, this study only confirms the effect of exosomes released by SiO_2_-stimulated macrophage on macrophage apoptosis and fibroblast activation. Furthermore, the role of exosomes in the occurrence and development of silicosis was proved. However, a large number of in-depth animal and cytological experiments are needed to verify the specific regulatory role of lncRNA MSTRG.43085.16.

## 5. Conclusions

In general, our study provided the expression profile of exosomal lncRNAs in the peripheral serum of silicosis patients. Moreover, bioinformatics analysis identified that the lncRNA MSTRG.43085.16/PARP1 pathway played a crucial role in the process of silicosis. Finally, macrophage-derived exosomes might contribute to affecting the process of silicosis by inhibiting the apoptosis of macrophages and promoting the activation of fibroblasts.

## Figures and Tables

**Table 1:** Basic Information of Silicosis Patients and Healthy Controls.

Variables	Patient	Control
N	20	29
Age ^a^	54.7 ± 2.4	54.7 ± 2.6
Accumulated time of exposure silica particle (years) ^b^	26.2 ± 4.3	32.3 ± 7.3
Stage I	20	—

^a^: The *t*-test was used to compare the mean of the patients and the control group (*t* = 0.053, *p* = 0.958). ^b^: The *t*-test was used to compare the mean between the patient and the control group (*t* = −3.296, *p* = 0.02).

**Table 2:** Differentially Expressed LncRNAs in Silicosis Patients.

Gene ID	Phenotype	*p*-Value	log2FC ^a^	Regulated
MSTRG.43086.12	Predicted	0.026033861	16.96111063	up
MSTRG.43085.16	Predicted	0.014004392	16.31897018	up
MSTRG.43086.7	Predicted	0.033170095	−17.98422883	down
MSTRG.43086.1	Predicted	0.003682449	18.46426007	up
MSTRG.43085.22	Predicted	0.033005847	−18.57420519	down
MSTRG.43085.26	Predicted	0.032206632	12.66049827	up
MSTRG.43085.13	Predicted	0.014999992	−16.92435452	down
FP236383.3-201	Predicted	0.006676377	−15.10674163	down

^a^: Fold change = Case mean/control mean. FC, fold change.

## Data Availability

The data that support the findings of this study are openly available in [NCBI] at https://dataview.ncbi.nlm.nih.gov/object/PRJNA825119?reviewer=i6hd0pn22ap39s80s8rfv18npj, reference number [PRJNA825119], accessed on 19 April 2022.

## Supplementary Materials

The following supporting information can be downloaded at: https://www.mdpi.com/article/10.3390/biom14020146/s1, Figure S1: The principal component analysis were used to investigate the coherence of the data distribution with the actual sample assignments (H, the healthy control; S, the patient). Table S1: KEGG pathway enrichment for the target genes of lncRNA MSTRG.43085.16. Table S2: Functional re-annotation for the target genes of lncRNA MSTRG.43085.16 based on the GO database.
