# Supplementary material for: lncRNA Profiling of Exosomes and Its Communication Role in Regulating Silica-Stimulated Macrophage Apoptosis and Fibroblast Activation
Source: Biomolecules. 2024 Jan 24;14(2):146. doi: 10.3390/biom14020146 (PMC10886698; doi:10.3390/biom14020146)
Supplement: Supplementary file 1 [file biomolecules-14-00146-s001.zip › supplementary-20231109.pdf]

title page

LncRNA profiling of exosomal and its communication role in regulating silica-stimulated macrophage apoptosis and fibroblast activation

Jiaqi Ban[1,3], Pengwei Ma[2], Shuai Chang[2], Xin Wang[4], and Fangwei Liu [1, 2*]

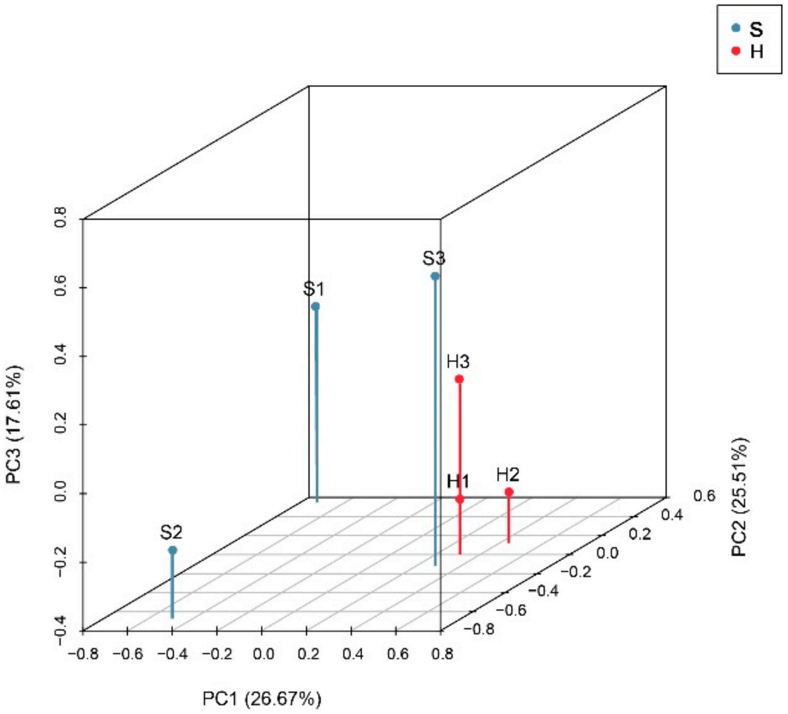

Supplementary figure S1

The principal component analysis were used to investigate the coherence of the data distribution with the actual sample assignments (H, the healthy control; S, the patient).

Table S1. KEGG pathway enrichment for the target genes of lncRNA MSTRG.43085.16

KEGG A class	KEGG B class	Pathway	Count	Pathway ID
Cellular Processes	Cellular community eukaryotes	Focal adhesion	79	ko04510
Cellular Processes	Cell motility	Regulation of actin cytoskeleton	77	ko04810
Cellular Processes	Cellular community eukaryotes	Signaling pathways regulating pluripotency of stem cells	59	ko04550
Cellular Processes	Transport and catabolism	Phagosome	57	ko04145
Cellular Processes	Cell growth and death	Cell cycle	55	ko04110
Cellular Processes	Transport and catabolism	Lysosome	54	ko04142
**Cellular Processes**	**Cell growth and death**	**Apoptosis**	**54**	**ko04210**
Cellular Processes	Cellular Processes	Endocytosis	110	ko04144
Environmental Information Processing	Signal transduction	MAPK signaling pathway	93	ko04010
Environmental Information Processing	Signal transduction	Rap1 signaling pathway	83	ko04015
Environmental Information Processing	Signal transduction	Ras signaling pathway	79	ko04014
Environmental Information Processing	Signaling molecules and interaction	Neuroactive ligand-receptor interaction	70	ko04080
Environmental Information Processing	Signal transduction	mTOR signaling pathway	64	ko04150
Environmental Information Processing	Signal transduction	cAMP signaling pathway	63	ko04024
Environmental Information Processing	Signaling molecules and interaction	Cytokine-cytokine receptor interaction	62	ko04060
Environmental Information Processing	Signal transduction	Phospholipase D signaling pathway	56	ko04072
Environmental Information Processing	Signal transduction	Hippo signaling pathway	56	ko04390
Environmental Information Processing	Signal transduction	cGMP-PKG signaling pathway	55	ko04022
Environmental Information Processing	Signal transduction	Calcium signaling pathway	55	ko04020
Environmental Information Processing	Signal transduction	Wnt signaling pathway	53	ko04310

Environmental Information Processing	Signal transduction	Sphingolipid signaling pathway	52	ko04071
Environmental Information Processing	Signal transduction	Phosphatidylinositol signaling system	51	ko04070
Environmental Information Processing	Signal transduction	TNF signaling pathway	51	ko04668
Environmental Information Processing	Signal transduction	FoxO signaling pathway	50	ko04068
Environmental Information Processing	Signal transduction	PI3K-Akt signaling pathway	121	ko04151
Genetic Information Processing	Folding, sorting and degradation	Protein processing in endoplasmic reticulum	67	ko04141
Genetic Information Processing	Transcription	Spliceosome	65	ko03040
Genetic Information Processing	Folding, sorting and degradation	Ubiquitin mediated proteolysis	64	ko04120
Genetic Information Processing	Transcription	RNA transport	61	ko03013
Human Diseases	Infectious disease: viral	Epstein-Barr virus infection	88	ko05169
Human Diseases	Cancer: overview	Viral carcinogenesis	87	ko05203
Human Diseases	Neurodegenerative disease	Huntington's disease	83	ko05016
Human Diseases	Cancer: overview	Proteoglycans in cancer	80	ko05205
Human Diseases	Endocrine and metabolic disease	Non-alcoholic fatty liver disease (NAFLD)	71	ko04932
Human Diseases	Infectious disease: viral	Herpes simplex infection	66	ko05168
Human Diseases	Neurodegenerative disease	Alzheimer's disease	65	ko05010
Human Diseases	Infectious disease: bacterial	Tuberculosis	65	ko05152
Human Diseases	Cancer: overview	Transcriptional misregulation in cancer	64	ko05202
Human Diseases	Neurodegenerative disease	Parkinson's disease	62	ko05012
Human Diseases	Cancer: overview	MicroRNAs in cancer	61	ko05206
Human Diseases	Infectious disease: viral	Influenza A	60	ko05164
Human Diseases	Infectious disease: viral	Hepatitis B	54	ko05161
Human Diseases	Infectious disease: viral	Measles	52	ko05162

Human Diseases	Substance dependence	Alcoholism	50	ko05034
Human Diseases	Cancer: overview	Pathways in cancer	159	ko05200
Human Diseases	Infectious disease: viral	HTLV-I infection	106	ko05166
Metabolism	Energy metabolism	Oxidative phosphorylation	60	ko00190
Metabolism	Nucleotide metabolism	Purine metabolism	56	ko00230
Organismal Systems	Immune system	Chemokine signaling pathway	69	ko04062
Organismal Systems	Development and regeneration	Axon guidance	67	ko04360
Organismal Systems	Development and regeneration	Osteoclast differentiation	65	ko04380
Organismal Systems	Endocrine system	Insulin signaling pathway	55	ko04910
Organismal Systems	Immune system	Platelet activation	52	ko04611
Organismal Systems	Nervous system	Neurotrophin signaling pathway	50	ko04722
Organismal Systems	Sensory system	Olfactory transduction	126	ko04740

a) Apoptosis pathway was highlighted in bold font; b) Only Pathways with a count value ≥ 50 were shown.

Table S2. Functional re-annotation for the target genes of lncRNA MSTRG.43085.16 based on the GO database

GOTerm	Ontology Source	p Value	FDR	Count	List Total	Ratio (%)	Associated Genes Found
Apoptosis	KEGG	2.63E-107	1.315E-105	60	63	93.75	CTSZ, PRF1, PIK3CD, PIK3CB, LMNB2, LMNB1, IKBKB, TUBA1C, CTSO, CTSL, CAPN2, CASP2, CAPN1, IKBKG, CTSD, DFFA, DAXX, MAP2K1, PARP4, ENDOG, MAP2K2, PARP1, APAF1, PDPK1, TRAF2, TNFRSF1A, PIK3CA, DDIT3, IL3RA, RAF1, TP53, BCL2A1, ITPR1, HTRA2, PIK3R3, CSF2RB, PIK3R1, SPTA1, MAPK8, BCL2L11, PMAIP1, RIPK1, FADD, BID, MCL1, MAPK3, BAD, GADD45A, DAB2IP, CFLAR, EIF2S1, NFKB1, NFKBIA, MAPK10, CYCS, BAX, KRAS, ATM, BCL2L1, TUBA8
NF-kappa B signaling pathway	KEGG	1.31E-11	1.31E-11	13	63	20.31	PARP1, BCL2A1, GADD45A, TRAF2, CFLAR, NFKB1, TNFRSF1A, NFKBIA, IKBKB, RIPK1, ATM, IKBKG, BCL2L1
Necroptosis	KEGG	2.49E-08	2.49E-08	12	63	18.75	MAPK10, MAPK8, PARP1, CAPN2, BAX, RIPK1, TRAF2, FADD, CFLAR, CAPN1, BID, TNFRSF1A
Diabetic cardiomyopathy	KEGG	2.07E-05	2.07E-05	10	63	15.63	MAPK10, MAPK8, PIK3CA, PARP1, PIK3R3, PIK3CD, PIK3CB, PIK3R1, CTSD, NFKB1
apoptotic process	Biological Process	7.51E-24	6.78E-21	27	62	42.19	BCL2A1, PRF1, BCL2L11, CASP2, PMAIP1, RIPK1, FADD, IKBKG, BID, MCL1, MAPK3, DAXX, PARP1, APAF1, GADD45A, BAD, TRAF2, CFLAR, NFKB1, TNFRSF1A, NFKBIA, DDIT3, BAX, CYCS, RAF1, TP53, BCL2L1
positive regulation of	Biologica	9.15E-06	2.12E-04	5	62	7.81	DAXX, PARP1, BAD, CAPN2, EIF2S1

neuron death	l Process						
positive regulation of transcription from RNA polymerase II promoter	Biological Process	1.63E-05	3.46E-04	15	62	23.44	PARP1, DAB2IP, PIK3R1, NFKB1, TNFRSF1A, NFKBIA, IKBKB, DDIT3, RIPK1, ATM, FADD, IKBKG, RAF1, TP53, MAPK3
cellular response to DNA damage stimulus	Biological Process	1.89E-05	3.80E-04	8	62	12.50	PARP4, PARP1, DDIT3, PMAIP1, ATM, IKBKG, TP53, MAPK3
cellular response to UV	Biological Process	2.51E-05	4.73E-04	5	62	7.81	PARP1, BAX, PIK3R1, TP53, EIF2S1
protein autoprocessing	Biological Process	8.26E-05	0.001223	4	62	6.25	PARP1, CTSL, CAPN2, HTRA2
response to gamma radiation	Biological Process	0.003758	0.032011	3	62	4.69	PARP1, BAX, TP53
double-strand break repair	Biological Process	0.00388	0.032743	4	62	6.25	MAPK8, PARP1, ATM, TP53
cellular response to oxidative stress	Biological Process	0.004448	0.035863	4	62	6.25	PARP1, HTRA2, CYCS, EIF2S1
cellular response to insulin stimulus	Biological Process	0.004937	0.039455	4	62	6.25	PARP1, PDPK1, CFLAR, PIK3R1

a) FDR, false discovery rate; Nr.Genes (%) = Number of genes of interest / Number of genes in total.
