# Supplementary figures and images for: lncRNA Profiling of Exosomes and Its Communication Role in Regulating Silica-Stimulated Macrophage Apoptosis and Fibroblast Activation
Source: Biomolecules. 2024 Jan 24;14(2):146. doi: 10.3390/biom14020146 (PMC10886698; doi:10.3390/biom14020146)

Original blot:

**Fig. 5E**

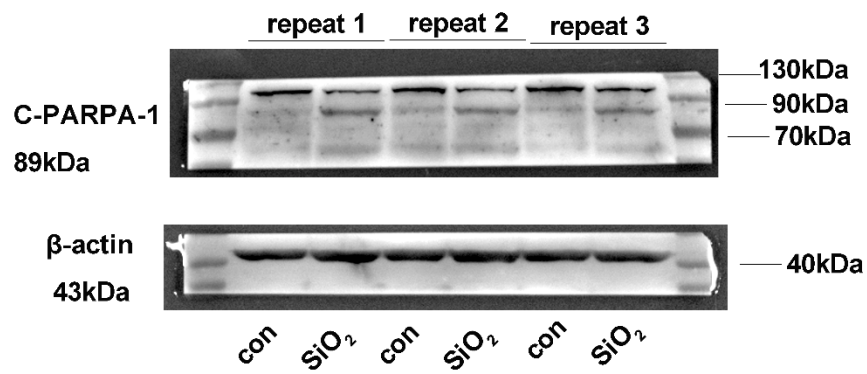

**Fig. 6A**

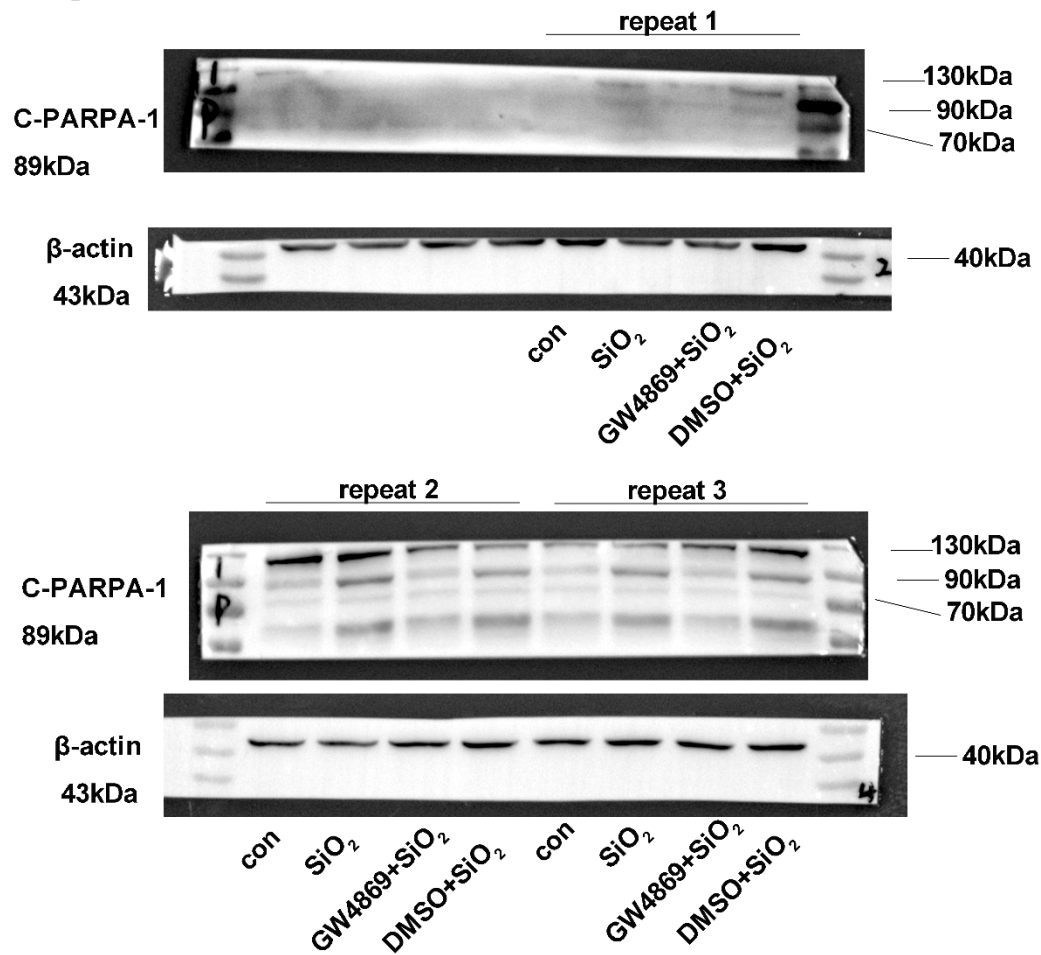

Supplement: Supplementary file 1 [file biomolecules-14-00146-s001.zip › Supplementary Materials- Original blot.pdf]
